# Molecular Basis for Chiral Selection in RNA Aminoacylation

**DOI:** 10.3390/ijms12074745

**Published:** 2011-07-22

**Authors:** Koji Tamura

**Affiliations:** 1 Department of Biological Science and Technology, Tokyo University of Science, 2641 Yamazaki, Noda, Chiba 278-8510, Japan; 2 Research Institute for Science and Technology, Tokyo University of Science, 2641 Yamazaki, Noda, Chiba 278-8510, Japan; 3 PRESTO, Japan Science and Technology Agency, 4-1-8 Honcho, Kawaguchi, Saitama 332-0012, Japan

**Keywords:** homochirality, amino acid, RNA, aminoacylation, stereochemistry, extended double helix, origin of life

## Abstract

The chiral-selective aminoacylation of an RNA minihelix is a potential progenitor to modern tRNA-based protein synthesis using l-amino acids. This article describes the molecular basis for this chiral selection. The extended double helical form of an RNA minihelix with a CCA triplet (acceptor of an amino acid), an aminoacyl phosphate donor nucleotide (mimic of aminoacyl-AMP), and a bridging nucleotide facilitates chiral-selective aminoacylation. Energetically, the reaction is characterized by a downhill reaction wherein an amino acid migrates from a high-energy acyl phosphate linkage to a lower-energy carboxyl ester linkage. The reaction occurs under the restriction that the nucleophilic attack of O, from 3′-OH in the terminal CCA, to C, from C=O in the acyl phosphate linkage, must occur at a Bürgi-Dunitz angle, which is defined as the O–C=O angle of approximately 105°. The extended double helical form results in a steric hindrance at the side chain of the amino acid leading to chiral preference combined with cation coordinations in the amino acid and the phosphate oxygen. Such a system could have developed into the protein biosynthetic system with an exclusively chiral component (l-amino acids) via (proto) ribosomes.

## 1. Introduction

A distinguishing characteristic of the biological system is that the building blocks are composed exclusively of homochiral molecules. Since the discovery of the double helical structure of DNA by Watson and Crick [1], the fundamental mechanisms of molecular biology have been elucidated. Crick named the sequential transfer of genetic information the central dogma [2]. DNA stores the genetic information in its nucleotide sequence, and proteins play important roles in catalyzing individual reactions. Although new roles of non-coding RNA have recently been discovered [3–5], the most well-known and primary function of RNA is to mediate the genetic information encoded in DNA in the central dogma: mRNAs are transcribed from DNA, after which amino acids are incorporated into proteins on ribosomes via aminoacyl-tRNAs, according to the nucleotide sequences found on the mRNAs [6,7].

It is noteworthy that natural proteins are composed of l-amino acids, whereas nucleic acids (RNA or DNA) are composed of d-sugars. Thus far, several explanations have been proposed regarding the origin of l-amino acids in biological systems. Some believe the origin lies in the physical properties of elementary particles [8,9], in the effect of circular polarized light in the universe [10], or in the enantioselective autocatalysis by chiral materials [11–13]. However, the formation of chiral components does not necessarily lead to the formation of a homochiral biological system. In the current biological system, tRNA aminoacylation is the step where RNA and amino acids specifically interact with each other; the correspondence between RNA codons and amino acids is known as the genetic code [14]. Homochirality and the genetic code might have been established simultaneously during the interaction of amino acids with trinucleotides [15]. In this article, I propose a possible scenario for the emergence of the homochirality of amino acids as the result of RNA aminoacylation, and explain its mechanism based on the stereochemistry of amino acids and RNA.

## 2. Chiral Selective Aminoacylation of RNA

**Figure 1:**
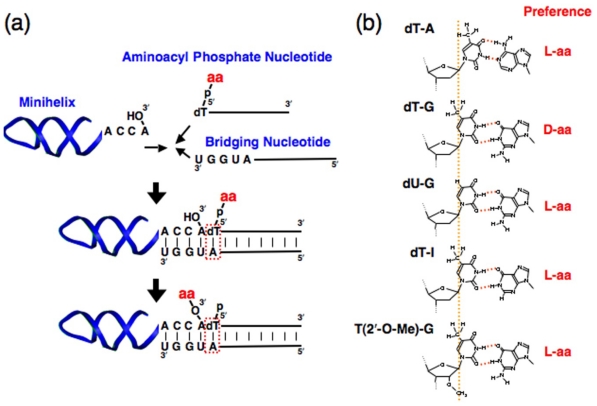
(**a**) Scheme for the chiral-selective aminoacylation of an RNA minihelix with an aminoacyl phosphate nucleotide in the presence of a bridging nucleotide; (**b**) A comparison of Watson-Crick (dT-A) and wobble base pairings (dT-G, dU-G, dT-I, and T(2′-O-CH_3_)-G) at the position closest to the amino acid-attachment site. The chiral preference for aminoacylation is indicated in red [20,21].

tRNA aminoacylation is catalyzed by aminoacyl tRNA synthetases and occurs via 2 consecutive reactions in biological systems: amino acid activation through the formation of aminoacyl-AMP and amino acid transfer to tRNA [14]. Based on the fact that aminoacyl-AMP and oligonucleotides could have been formed in prebiotic conditions [16,17], a system for the non-enzymatic aminoacylation of the RNA minihelix (progenitor of modern tRNA) was developed. The minihelix corresponds to the acceptor stem and TΨC stem/loop of tRNA [18]. The non-enzymatic aminoacylation system is composed of 3 molecules: an RNA minihelix (amino acid acceptor), an aminoacyl phosphate nucleotide (amino acid donor), and a bridging nucleotide. These molecules constitute an extended double-helix structure, and all of the base pairings hybridizing each chain are of the Watson-Crick type. The CCA end (aminoacylation site) and the aminoacyl phosphate are placed in close proximity by a bridging nucleotide (Figure 1a). Notably, the system exhibited chiral-selective (l-amino acid preference) aminoacylation, and approximately 15% of the minihelix was aminoacylated in the case of l-Ala [19].

Aminoacylation occurred site-specifically, and the aminoacylation site was restricted to 3′-OH, not 2′-OH, irrespective of the chirality of amino acids. The chiral selectivity would not be dependent on the bulkiness of the amino acid-side chains; at least in the case of several amino acids with different degrees of bulkiness, *i.e.*, Ala, Leu, and Phe, l-amino acids were almost equally aminoacylated with approximately 4-fold higher efficiency than d-amino acids, regardless of the differences in the side chains [19].

This finding is consistent with the RNA world hypothesis [22]. Because proteins are used to generate other proteins in the modern biological system, the ultimate question is “How was the first protein produced?” The discovery of “ribozyme” could solve the so-called chicken-or-egg conundrum of whether nucleic acids or proteins originated first. RNA is now thought to have functioned as both a carrier of genetic information and a catalyst in the primitive stage of life [23,24], leading to the concept of an RNA world [22]. It is believed that the RNA world was followed by a protein world, and the aminoacylation of primitive tRNA would have been a critical bridge connecting the 2 worlds [18]. Thus, tRNAs charged with l-amino acids could form proteins composed of l-amino acids by using proto-ribosomes much the same as modern proteins. In this context, the chiral-selective aminoacylation of RNA is a crucial development in consideration of the origin of amino acid homochirality in biological systems [19].

The putative evolutionary story is described in detail in the literature [25–28], and therefore, here, I would like to focus on the molecular mechanism determining the chiral selectivity of the reaction.

## 3. Chemical Features of Aminoacylation Reactions

In the modern system, each aminoacyl tRNA synthetase catalyzes an amino acid-specific aminoacylation reaction [14]. The enzymes are grouped into 2 classes based on the structure of the catalytic core [29]. However, in the simplified model of the chiral-selective aminoacylation of RNA proposed by Tamura and Schimmel [19], RNA aminoacylation is performed using a high-energy aminoacyl phosphate donor nucleotide (mimic of aminoacyl-AMP) with the help of a bridging nucleotide. Chemically, aminoacylation is characterized by a downhill reaction, beginning with a high-energy aminoacyl phosphate linkage and forming a lower-energy aminoacyl carboxyl ester linkage [30]: the free energy of hydrolysis of an aminoacyl phosphate is approximately 3 kcal/mol greater than that of an aminoacyl ester. The rationale is equally true in both the modern aminoacylation by aminoacyl tRNA synthetases and the Tamura-Schimmel system.

**Figure 2:**
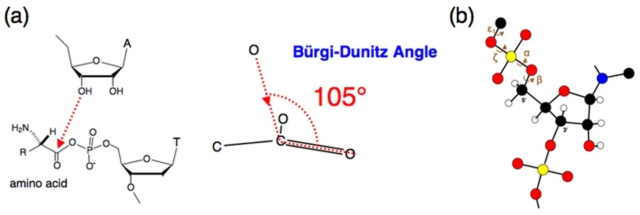
(**a**) A close look at the nucleophilic attack of the 3′-O to the carbonyl carbon of the acyl phosphate group. The reaction is restricted at the Bürgi-Dunitz angle [31], which is defined as the O–C=O angle of approximately 105°; (**b**) A polyribonucleotide and the torsion angles around a 5′-phosphate. Black, carbon; white, hydrogen; red, oxygen; blue, nitrogen and yellow, phosphorus.

In the model reaction, the oxygen from the 3′-OH of the terminal adenosine of the minihelix approaches the carbonyl carbon at the Bürgi-Dunitz angle, which is defined as the O–C=O angle of approximately 105° (Figure 2a) [31]. In this sense, the direction of the approach is not random, but is restricted. Such a restriction is important for determining chiral selectivity.

The reaction is characterized by a typical nucleophilic attack. It goes through the tetrahedral intermediate formation and finally produces a 3′-aminoacyl-minihelix. Analysis by molecular dynamics revealed that the product analogs (3′-l-alanyl-adenosine *vs.* 3′-d-alanyl-adenosine) exhibit no critical differences in free energy [32], suggesting that the reaction proceeds under kinetic control and not thermodynamic control.

An amino acid is attached via an acyl phosphate linkage, which is a single bond, and the rotation around the bond is not strongly restricted. In the case of dinucleotide monophosphate and the nucleotides composed of more components, steric restrictions are also observed around the phosphate: torsion angles α, β, ɛ and ζ (Figure 2b) are restricted to be −*sc* (*synclinal*), *ap* (*antiperiplanar*), *ap* (*antiperiplanar*) or −*ac* (*anticlinal*), and −*sc* (*synclinal*) or −*ap* (*antiperiplanar*), respectively [33]. However, the rotations around the phosphate (α, β, ɛ, ζ) of the 5′-aminoacyl phosphate nucleotide, which is used as a donor in non-enzymatic aminoacylation reactions, can be treated in the same way as those around the C–C single bond of ethane. That is, the rotations around the phosphate are not restricted, and the staggered and eclipsed conformations are relatively freely changeable. Thus, the effect of potential differences in conformational preference based on trans-gauche isomerizations [34] could be ruled out because the free-energy barrier between these conformers would be around 3 kcal/mol [35], which can be overcome by the thermal energy of the molecules in the experimental condition (0 °C). Therefore, the positioning of the amino group, hydrogen atom, and side chain, which are all connected to the alpha carbon of the amino acid, should not be restricted in terms of the free energy of the structures of the naked conformers.

Considering these circumstances, here, I will present a possibility that could explain the chiral selectivity of the reaction. Metal ions play important roles in biological systems, and this also holds true in this simple model system. The model system contains Na^+^ and Mg^2+^. The 3 components of the experimental system—an RNA minihelix, an aminoacyl phosphate nucleotide, and a bridging nucleotide—interact each other through Watson-Crick base pairing to form an extended double helical structure. Na^+^ is known to strengthen the base pairing [36], and Mg^2+^ is often crucial in RNA chemistry [37]. Because the different concentration of these ions could affect various aspects of the reactions, *i.e.*, metal ion binding ability, base pairing strength, and conformational differences dependent on metal concentration, constant concentrations of Na^+^ (1000 mM) and Mg^2+^ (10 mM) were used in this model system [19], although the effect of the differences in ion concentrations must be investigated extensively in the future from many different experimental standpoints.

**Figure 3:**
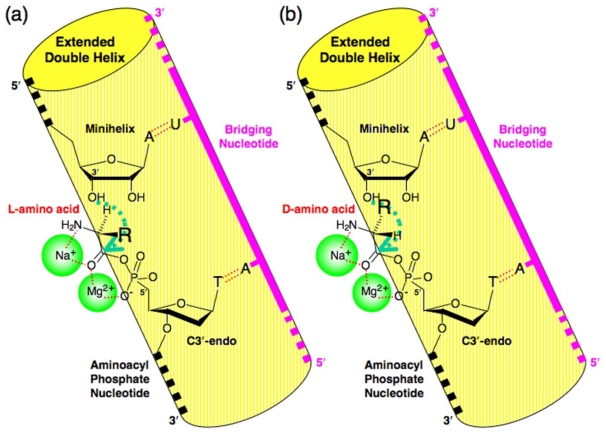
Possible positionings of (**a**) l-amino acid and (**b**) d-amino acid with respect to the 3′-OH of the minihelix in the extended double helical conformation. Na^+^ and Mg^2+^ are thought to coordinate in bidentate mode and affect the amino acid conformation in a sterical sense. The green arrows indicate the nucleophilic attack of the 3′-O of the minihelix. The yellow cylinders are the schematic representation of the extended double helix composed of 3 molecules (an RNA minihelix, an aminoacyl phosphate nucleotide, and a bridging nucleotide).

In the model system, Na^+^ is thought to be complexed with amino acids between the carbonyl and amino groups in a bidentate mode (Figure 3). This situation is highly probable because the *ab initio* calculations have also revealed that the most stable isomer of the glycine-Na^+^ complex is produced in this mode [38–40]. Further evidence for bidentate coordination of Na^+^ between the 2 carbonyl O-atoms of *N*-acetyl-amino acid has been proposed [38], and non-enzymatic aminoacylation experiments using Ala and *N*-acetyl-Ala attached to oligonucleotides via acyl phosphate bonds revealed the same chiral selectivity (l-amino acid preference) [20]. This coordination of Na^+^ confirms that the amino group is located outside of the extended double helix composed of the CCA, donor nucleotide and bridging nucleotide (Figure 3). In addition, Mg^2+^ is believed to be coordinated between the carbonyl oxygen and the phosphate oxygen, as shown by the NMR analysis of ATP in the presence of Mg^2+^ [41] and also by the X-ray crystallographic structure of the Mg^2+^-coordinated dNTP complexed with DNA polymerases [42,43].

The atomic radius of Na^+^ and Mg^2+^ is 1.86 Å and 1.60 Å, respectively. In this situation, the sequential coordination of cations could cause the associated regions to be placed outside of the double helical structure to avoid their steric interference with the inner region of the helix (Figure 3). Such requirements are important to the structural basis for chiral preference during the reaction. Thus, the side chain of an l-amino acid would be distal to the 3′-OH of the terminal adenosine of the RNA minihelix, whereas that of a d-amino acid would be located close to the 3′-OH (Figure 3) [20,21].

Na^+^ and Mg^2+^ can behave as counter ions for the phosphate anions of nucleic acids due to their electrical charge. NMR analysis clearly indicates that a divalent cation has greater binding strength with the phosphates of ATP than a monovalent cation [44]. This is true because Mg^2+^ coordinates between oxygen atoms accompanied by changes in the O–P–O angle that also strengthen the binding of the cation with the phosphates [45], whereas Na^+^ coordinates directly with the phosphates of the nucleotides [44]. Therefore, even with such differences in the experimental concentrations of Na^+^ and Mg^2+^, Mg^2+^ could specifically coordinate between the carbonyl oxygen and the phosphate oxygen.

## 4. Proof of the Steric Effect Caused by the Positioning of Amino acid Side Chains in Aminoacylation

Local conformation is a crucial factor in determining the chiral selectivity of a reaction. This is especially true for the conformation closest to the 3′-terminal adenosine (acceptor site) and the 5′-terminal aminoacyl phosphate nucleotide (donor site). The aminoacylation system is composed of 3 molecules (an RNA minihelix, an aminoacyl phosphate nucleotide, and a bridging nucleotide) in the form of an extended double helix. Each part of the helix interacts according to the Watson-Crick manner of base pairings. Watson-Crick base pairings are fundamental to the transfer of genetic information that most often occurs during DNA replication. However, Watson-Crick type base parings are not the only base-pairing interactions observed in biological systems. Other base-pairings appear in the structures and interactions especially of RNA. Among them, the wobble interaction occurs during codon-anticodon base pairings in mRNA-tRNA interactions, where G can be paired not only with C, but also with U [46]. The wobble base pairing, G–U, twists the double helix as compared to Watson-Crick base pairings, because the amino group at position 2 of G protrudes into the minor groove side, and the NH at position 1 and C=O at position 6 of G are hydrogen bonded with the C=O at position 2 and NH at position 3 of U, respectively.

In the original reaction, amino acid was attached to the 5′-phosphate group of deoxythymidine (Figure 1a). Thymine has a methyl group at position 5, and this group can be a good indicator of the local conformation of the double helical region by monitoring its steric hindrance interaction with the side chain of both l- and d-amino acids. Interestingly, introduction of the dT-G wobble base pair at the position closest to the acceptor RNA reduces the yield of the l-aminoacyl-minihelix (Figure 1b) [20]. However, substitutions of dU-G or dT-I (I: inosine) did not affect the reaction and retained the original chiral (l-amino acid) preference (Figure 1b) [20]. dU lacks the methyl group at position 5 as compared to dT, and I lacks the amino group at position 2 as compared to G. The differences in aminoacylation preferences should be interpreted in terms of such structural differences in the nucleotides (Figure 1b).

The interactions between the side chains of the l-amino acids and the methyl group of thymidine are found to be different when dT-G and dT-I are compared. Both dT-G and dT-I are wobble base pairs, and their helix ends are distorted similarly as compared to Watson*-*Crick type base pairing (dT-A). Therefore, the differences in chiral preference are believed to be derived from the puckering differences of dT between dT-G and dT-I (Figure 1b).

**Figure 4:**
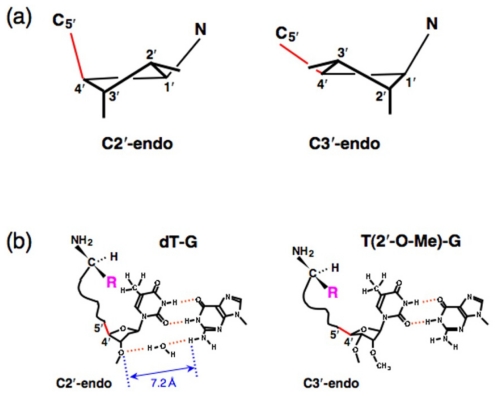
(**a**) Schematic representation of the typical puckerings of the ribose ring [33]; (**b**) The comparison of the puckering effects in the case of dT-G and T(2′-O-CH_3_) [21]. The distance between O (of 3′-OH) and H (of the amino group) at position 2 of G in the case of dT-G is expected to be approximately 7.2 Å [50]. The C2′-O-CH_3_ group stabilizes the C3′-endo conformation [49].

The atoms composing the 5-membered furanose ring are not positioned flat. The atom positioned on the same side of C5′ is termed “endo,” and that on the opposite side is termed “exo” (Figure 4a) [33]. By using these terms, the puckering of the sugar can be generally defined. There are 2 major puckering modes in ribose, C2′-endo and C3′-endo (Figure 4a). In the case of tRNA, it is well known that xo^5^U at the first letter of the anticodon stabilizes the C2′-endo conformation, whereas xm^5^U stabilizes the C3′-endo conformation, both of which are critical for retaining translational fidelity [47,48]. Similarly, the C2′-O-CH_3_ group stabilizes the C3′-endo conformation (Figure 4b) [49]. Surprisingly, in the case of T(2′-O-CH_3_)-G, aminoacylation of the minihelix showed an l-amino acid preference, which is in contrast to what is observed in the case of dT-G (Figure 1b). These different preferences are attributable to the different puckerings of dT-G (C2′-endo) and T(2′-O-CH_3_)-G (C3′-endo) (Figure 4b) [21].

When considering the position of the amino acid in the model reaction, a critical difference that results from different sugar puckerings is observed. In a C2′-endo conformation, the C4′–C5′ bond of the ribose is positioned “straight up,” but in a C3′-endo conformation, the same bond is positioned “lying down” (Figure 4a) [33]. These differences could affect the position of the amino acid connected through an aminoacyl phosphate linkage at the 5′-end of the oligonucleotide. Steric hindrance may have occurred in the case of dT-G, but not in the case of T(2′-O-CH_3_)-G. The possible conformation is that the side chain of l-amino acids is positioned close to the protruding methyl group of dT, and the same side chain is positioned far from the methyl group of T(2′-O-CH_3_) (Figure 4b). This interpretation is consistent with the results of aminoacylation (Figure 1b). Thus, dT-G is believed to possess the C2′-endo conformation. Previous structural analysis by NMR clearly showed that the distance between the 3′-O of dT and the H of the amino group at position 2 of G in a dT-dG double helix is 7.2 Å [50], which is the perfect distance required to form 2 hydrogen bonds with a water molecule. Such hydrogen bonds are likely to stabilize the C2′-endo conformation in the case of dT-G (Figure 4b).

In the experiment using the mutants with wobble base pairing, the results excluding those for dT-G demonstrated that l-aminoacyl-minihelix formation is preferred over d-aminoacyl minihelix formation with similar quantitative effects (approximately 4-fold difference) to the original construct with a Watson-Crick pair (dT-A) at the position [20]. Importantly, in the case of dT-G, the d-amino acid preference results from the decrease in l-aminoacyl-minihelix formation without altering the production of the d-aminoacyl-minihelix [20]. This fact, combined with the results for both dT-G and T(2′-O-CH_3_), clearly suggest that the decrease of l-aminoacyl-minihelix formation in the case of dT-G was caused by pucker-dependent steric interference between the side chain of the amino acid and the methyl group of dT (Figure 4b).

Although the mutational study was performed using a 5′-aminoacyl phosphate deoxyribo-oligonucleotide and not a ribo-oligonucleotide, the RNA-DNA hybrid formed with the bridging oligonucleotide is expected to have the geometry of an A-form helix [51], as observed in RNA-RNA duplexes. In addition, the same chiral selectivity (l-amino acid preference) was also detected using a 5′-aminoacyl phosphate ribo-oligonucleotide. Therefore, the results using deoxynucleotides for the aminoacyl phosphate oligonucleotide are general, and the use of deoxynucleotides is valid for reasons of technical convenience.

The original chiral-selective aminoacylation system consisting of an extended double helix structure with 3 components uses only Watson-Crick type of base-pairings. However, this feature is not specific to this model; it was probably adopted by biological systems during evolution. The stability imparted by the stacking of 2 wobble G-U base pairs is known to be lower than that imparted by the stacking of 2 Watson-Crick base pairs, according to the free energy analysis [33,52]. Therefore, thermodynamically, a construct having an extended double helix structure with 3 components must be created by using Watson-Crick type base pairings, similar to the chiral-selective reaction model [19].

## 5. Conclusions

In spite of the evidence that shows the enantiomeric formation of amino acids in physical and chemical environments, there is no clear connection between enantiomeric excess and the homochirality of biological systems. tRNA aminoacylation is the unique reaction that connects amino acids and their nucleotide sequences, and this is related to the origin of the genetic code and life itself. The chiral selectivity of amino acids by nucleotides has been reported previously [53,54]. RNA-amino acid interactions are quite important when considering the origin of the genetic code, and a body of experimental data exploring this interaction has been obtained [55–63]. The evidence that l-amino acids select for l-amino acids during polymerization in the absence of RNA [64,65] suggests that a similar mechanism exists for the RNA-selection of l-amino acids. However, the chiral-selective aminoacylation of RNA in the model proposed by Tamura and Schimmel [19] is quite notable, because it can be associated with the formation of proteins with chiral components (l-amino acids) via (proto) ribosomes. In addition, an important feature of aminoacylation is that the reaction occurs under several restrictions forced by the extended double helix conformation. As described, steric hindrance of the side chain of the amino acid could be caused by the extended double helix conformation, possibly combined with coordinated cations.

The minihelix is considered to be the primitive form of modern tRNA, but presumably, the minihelix itself evolved from much smaller RNA fragments such as the microhelix, which is composed of only the acceptor stem of tRNA [18]. Definitively, the length of the helix required for chiral-selective aminoacylation would be much shorter than that of the minihelix because the point is whether the system can consist of an extended double helix structure using Watson-Crick type base pairing. This feature would be closely related to the origin of single-stranded NCCA sequences in tRNA and to the evolutionary pathway that led to the foundation of the aminoacylation system itself.
